# In vivo assessment of anticoagulant and antiplatelet effects of *Syzygium cumini* leaves extract in rabbits

**DOI:** 10.1186/s12906-019-2661-y

**Published:** 2019-09-03

**Authors:** Ahad Abdul Rehman, Azra Riaz, Muhammad Arif Asghar, Muhammad Liaquat Raza, Shadab Ahmed, Kamran Khan

**Affiliations:** 1 Department of Pharmacology, Faculty of Pharmacy, Jinnah Sindh Medical University, Rafiqui H.J Shaheed Road, Karachi, 75510 Pakistan; 2 Department of Pharmacology, Faculty of Pharmacy, University of Karachi, Karachi, Pakistan; 3 Department of Pharmaceutics, Faculty of Pharmacy, Jinnah Sindh Medical University, Rafiqui H.J Shaheed Road, Karachi, 75510 Pakistan; 4 Department of Clinical Sciences, Faculty of Medicine, Lund University Hospital, Lund, Sweden

**Keywords:** *Syzygium cumini*, Coagulation, Platelets, Thrombin, Prothrombin

## Abstract

**Background:**

*Syzygium cumini* (L.) Skeels. is one of the very popular traditionally used medicinal plants with numerous pharmacological activities including antioxidant, hypoglycemic and anti-inflammatory. However, actions of *S. cumini* on blood coagulation and other parameters of blood were poorly pharmacologically studied. Therefore, aim of this present investigation is to examine the effects of methanolic extract of *S. cumini* on blood coagulation and anticoagulation factors in healthy white albino rabbits at different doses.

**Methods:**

Blood samples were drawn twice during this study and biochemical assays were performed to determine the effect on different parameters such as coagulation, anticoagulation, hematological, Protein C (PC) and thrombin antithrombin (TAT) complex and platelet aggregation.

**Results:**

The results showed significant increase in RBCs, hemoglobin, hematocrit and platelets counts up to 1.4 × 10^3^/cm, 2.2 g/dl, 6%, 248.2 × 10^3^/cm respectively. While, thrombin and bleeding time were also prolonged in dose dependent manner which is highly significant (*p* ≤ 0.005) as compared to control. Similarly, highly significantly increased (*p* ≤ 0.005) in levels of protein C, thrombin antithrombin complex at dose of 500 mg/kg were observed. Whereas, levels of platelets aggregation and fibrinogen were decreased at high doses.

**Conclusion:**

The obtained findings of hematological and coagulation tests concludes possibly *S. cumini* possess anticoagulant and antiplatelet effects.

## Background

Human beings are using plants as medicine since centuries. Biological activities present in these plants and their derivatives are of great importance in healthcare history. Studies suggested that 30% of the all recent drugs are derived products of different plant species [1]. According to a survey conducted by World Health Organization (WHO), 80% of human population is largely dependent on plant sources for treatment of minor ailments. Recently, demand and importance of medicinal plants is increasing day by day due to its wide acceptance worldwide [2].

Plants and their derived products provides vital source, on which healthcare system of prehistoric population largely depends. Now a day, demand of new chemical moiety for the improvement of healthcare system is greatly supported by plant resources [3]. Studies showed that complementary and alternative medicine (CAM) also contributes their significant part in the treatment of cardiovascular related health problems, while herbal products are the most frequent part of CAM treatment [4].

Hemostasis is a normal physiological phenomenon in our internal body’s defense system that prevents loss of blood after hemorrhage, which converts blood into thick jell like mass at point of injury/damage. Blood coagulation is one of the steps in hemostasis mechanism which is responsible for the formation of blood clot that restricts flow of blood at site of injury. The mechanism of coagulation is a multi-step process involving activation of various enzymes which ultimately use its specific substrate and converts it into active enzyme [5]. Such proteolytic cascade proceeds till the formation of thrombin. Newly formed thrombin then breaks down soluble fibrinogen into insoluble fibrin which results in the formation of blood clot. Any inappropriateness in coagulation cascade may lead to pathological conditions i.e. venous thrombosis [6]. Atherosclerosis and several cardiovascular diseases can also be prevented by reducing thrombus formation and inhibition of coagulation cascade [7].

*Syzygium cumini* belongs to *Myrtaceae* family. It is widely distributed throughout the South Asian continent including Pakistan, India, Indonesia and Sri-Lanka. Chemically, leaves of *S. cumini* mainly contain high percentage of flavonoids i.e. ferulic acid, rutin and catechin while limonene found as major constituent of its essential oil [8].

Traditionally, *S. cumini* is reported in the treatment of ulcers, biliousness, bronchitis, dysentery, diabetes and its related complications. Moreover, leaf extract of *S. cumini* showed antimicrobial, anti-inflammatory, anti-nociceptive, antioxidant, antihyperlipidemic and cardioprotective potentials [9–13]. Cardioprotective potential of *S. cumini* in oxidative stress induced cardiac myocytes and protective effect in isoproterenol-induced myocardial infarction has also been reported [14]. Myricetin, catechin, and rutin present are common flavonoid found in the methanolic leaf extract of *S. cumini* possess a potent antioxidant activity [15]. Moreover, Mohamed et al. also reported α-pinene, camphene, myrcene, β-pinene and limonene are also responsible for antioxidant activity [16]. These polyphenolic compounds are effective due to free radical scavenging property along with their effects on cell signaling cascades and gene expression which would be helpful in increased synthesis of cells that leads to improve the blood cells count [17]. Different animal models have been used in thrombosis and atherosclerosis research studies, but no model completely mimics the disorders found in humans. Moreover, to our knowledge, only in vitro studies are conducted related to the development of atherosclerosis with one was having same methodology on *Punica granatum* [18]. Large animal models with more blood volume have, however, demonstrated a better suitability for translation to humans. Hence, rabbits were used in the current study because direct transformation from mice to humans may result in species-related differences. There is no previous report on anticoagulation and antiplatelet activities of *S. cumini* in literature to the best of our knowledge. Therefore the present study was conducted to evaluate the activity of *S. cumini* extract on blood coagulation and different blood parameters using rabbit’s model.

## Materials and methods

### Animals

This study was conducted on sixty healthy white male albino rabbits which were purchased from animal house of Dow University of Health Sciences, (DUHS), Karachi with average body weight of 1250 ± 50 g. Rabbits were chosen due to larger volume of blood was required to perform blood coagulation test and other parameters of study Animals were kept under prerequisite temperature of 23 °C ± 2 °C with humidity 50–60% in a light-dark cycle of 12 h each. Both control and tested animals were kept in same environment and provided with the standard food. Steel bottom cages were used to keep each rabbit separately with free access of food and water.

### Ethics and consent

Use of rabbits in this study was in accordance with the National Advisory Committee for Laboratory Animal Research guide for the care and use of Laboratory Animals and under the acquiescence of the Board of Advance Studies and Research, University of Karachi, Pakistan. The written consent form was also filled by the in-charge of the animal house (DUHS) to use these animals for our study. The study was approved by local research ethics committee of Board of Advance Studies and Research, University of Karachi, Pakistan and also approved by research review committee of Ziauddin Hospital Karachi-Pakistan with the reference number of 27/02/KK/ZHC.

### Plant material and extraction

Fresh leaves of *Syzygium cumini* (L.) Skeels. were purchased in the month of July from local commercial herbal research market of Lyari town, Karachi, Pakistan. It was identified by the meritorious Professor and Pharmacognosist Prof. Dr. Mansoor Ahmed at Center of Plant Conversation, University of Karachi, Pakistan with G.H. No. 94236. Leaves were washed using fresh water and allowed to air dry at room temperature. Then, grind by electrical blender. Approximately 200 g of powdered material was soaked in 500 mL of methanol (HPLC grade) for 2 weeks in a static condition. Separation of plant material from methanol was done through filtration using Whatman no. 1 filter paper, and then filtrate was concentrated using a rotary evaporator (IKA, Germany) at 40 °C under reduced pressure [19]. The final product was a dark brownish sticky mass weighing 7.3 g, which represents a yield of 3.65%.

### Drug treatment

Animals were divided into five groups with 10 rabbits in each group. 1st group received distilled water and served as control group for 2 months; 2nd and 3rd groups were administered orally with *S. cumini* leaves extract using two different doses i.e. 150 mg/kg and 500 mg/kg once daily. Whereas, 4th and 5th groups, served as standard drug’s group and received aspirin and warfarin respectively. Duration for warfarin administration was 6 days i.e. 3 days, initial dose used was 5 mg/kg, however, during later 3 days, 10 mg/kg dose was given. Aspirin was given at the dose of 150 mg/kg, once in a day for 6 days/week. Blood samples were drawn prior to 6 h fasting (avoid lipemia) at day 30th and 60th respectively, in ethylene diamine tetra acetate (EDTA) and trisodium citrate containing tubes and gel tubes from most dominant ear vein of rabbit [18]. Rabbits were humanely euthanatized by administration of Beuthanasia solution (100 mg/ml perntabarbitol, 1 ml/10 lbs) intravenously in the ear. Prior to euthanasia, each rabbit was sedated with an intramuscular injection of medetomidine (0.5 mg/kg) and ketamine (10 mg/kg).

### Hematological parameters

Following blood parameters were studied using hematology analyzer (Beckman Coulter, Inc. U.S). RBCs (Red blood cells), WBCs (White blood cells), platelets count, Hb (Hemoglobin concentration), MCH (Mean corpuscular hemoglobin), HCT (Hematocrit), MCV (Mean corpuscular volume), MCHC (Mean corpuscular hemoglobin concentration) and red cell distribution width.

### Bleeding time estimation

Bleeding time in studied animals was estimated according to the modified method [20]. Briefly, a minor incision (5 mm long and 1 mm deep) was made in the central artery of ear after removal of hairs. The slit opening was dried using blotting paper after every 30 s till the bleeding was stopped.

### Estimation of fibrinogen levels, thrombin, prothrombin and activated partial thromboplastin time

Trisodium citrate tubes were used to separate plasma from blood samples by centrifugation at 2000×*g* for 10 min. Humaclot duo coagulometer and human reagent kits (Huma count, Germany) were used to perform these tests. Clot formation was evaluated by turbidimetric technique, and changed in optimal plasma density was used to determine coagulation endpoint. Thrombin reagent (100 μL) was mixed with 200 μL plasma for determination of thrombin time (TT). While, prothrombin time (PT) was determined by adding 200 μL of thromboplastin reagent in 100 μL of plasma. The mixture was then incubated with aPTT-EL reagent (100 μL) at 37 °C for 15 min, further CaCl_2_ was added to determine activated partial thromboplastin time (aPTT). The level of fibrinogen was estimated using the method as described by McNerlan *et at.* [21].

### Platelets aggregation test

To assess platelet function, Agg RAM aggregometer (Helena Biosciences, Europe) was used, manufacturer instructions were followed. Blood was taken in the ratio of 9:1 v/v in trisodium citrate (3.8%). This analysis is necessary the use of platelets poor plasma (PPP). Therefore, blood samples were centrifuged for 14 min at 100×*g* and residual blood for 15 min at 1800×*g* in 14 K Humax to form platelets rich plasma (PRP) and PPP respectively. PRP prepared samples were standardized (approx. 250,000/mm^3^) with autologous PPP as needed [22]. Reactivity of platelets was evaluated at 37 °C for 10 min adopting method of Jeong et al. [23]. PRP absorbance denotes 0% aggregation and PPP absorbance denotes as 100% aggregation. Platelet aggregation was induced by arachidonic acid (AA), adenosine diphosphate (ADP), collagen and epinephrine. PRP (450 μL) was taken into cuvettes for incubation, while, PPP cuvette set to 100% aggregation using aggregating reagent. Transmittance was used for the determination of resultant aggregation and was expressed as %.

### Protein C (PC) and thrombin antithrombin (TAT) complex

PC and TAT complex kits of Elisa (Biotech Ltd. India) were used to determine the activity of protein C and thrombin-antithrombin complex. Absorbance of standard plasma was used as standard curves for PC and TAT complexes at 450 nm. However, activity of PC and TAT complexes in samples were referred as standard plasma activity. The guidelines of National Committee for Clinical Laboratory were followed for all estimations [24].

### Phytochemical characterization

Different standard chemical methods were utilized to determine the chemical composition in *S. cumini* methanolic leaves extract. Phytochemicals including flavonoids, phenolic compounds, terpenoids, tannins and alkaloids were screened using different identification tests i.e. Shinoda test, Ellagic Acid Test, Salkowski test, Ferric Chloride Test and Mayer’s test respectively [25].

### Statistical analysis

Data was represented as mean with standard error (SEM) and was analyzed using SPSS version 23 (IBM, USA). ANOVA with post-hoc analysis was utilized for values comparisons with control. *P*-values at level of < 0.05 & *P* < 0.005 were considered as significant and highly significant respectively.

## Results

Overall animal’s health evaluation such as average weight variation, skin ulceration, loss of activity, diarrhea, hematuria, salivation, tremor, vomiting, edema and aggressive behavior were observed in all control and test groups before and during the total period of experiment.

Table 1 reveals the effect of *S. cumini* on bleeding time (BT), Thrombin time (TT), Prothrombin time (PT), activated partial thromboplastin time (aPTT) and Fibrinogen (Fb) levels. At day 30th and 60th, there was highly significant increase in BT, while, decrease in Fb levels at both doses of *S. cumini* respectively. Significant increase in aPTT was observed by SCD1 and SCD2, whereas, PT was increased at 60th day in SCD2 group. Changes in TT were not significant after 30 and 60 days at both the doses as compared to the control group.

**Table 1:** In vivo comparison of *Syzygium cumini* leaves extract, warfarin and control on rabbit’s coagulation parameters. Humaclot duo coagulometer and human reagent kits were used to perform these tests. Clot formation was evaluated by turbidimetric technique

Groups	Days	Parameters				
		BT (Sec)	TT (Sec)	PT (Sec)	aPTT (Sec)	Fb (mg/dl)
Control	30	180.8 ± 36.82	20.6 ± 3.36	12.7 ± 0.85	14.6 ± 1.57	401.6 ± 24.66
	60	170.8 ± 19.38	21.2 ± 2.77	12.3 ± 1.05	14.2 ± 0.92	410.6 ± 17.79
SCD1	30	337.2 ± 11.69**	22.6 ± 2.61	17.4 ± 3.32	19.1 ± 1.69*	331.6 ± 13.72**
	60	341.2 ± 7.39**	23.0 ± 2.00	14.22 ± 1.54	17.9 ± 2.10*	320.2 ± 29.12**
SCD2	30	315.0 ± 66.33**	26.8 ± 1.92	24.4 ± 2.30	19.3 ± 3.45*	316.0 ± 23.45**
	60	303.0 ± 44.38**	24.8 ± 2.73	17.2 ± 2.19**	17.6 ± 2.07*	326.6 ± 26.26**
Warfarin	─	335.4 ± 24.60**	31.8 ± 4.97**	64.4 ± 17.21**	30.1 ± 1.30**	303.0 ± 21.54**

*n* = 10, Average values ± SEM,

*p ≤ 0.05 significant as compared to control, **p ≤ 0.005 highly significant as compared to control

SCD1: *S. cumini* dose 1; i.e. 150 mg/kg, SCD2: *S. cumini* dose 2; i.e. 500 mg/kg

*BT* Bleeding time, *TT* Thrombin time, *PT* Prothrombin time, *aPTT* activated partial thromboplastin time, *Fb* Fibrinogen level

Table 2 displays the effect of *S. cumini* extract on hematological parameters. High doses of extract showed significant increase in Hb and RBC count at day 30th when compared with control group, whereas, significant and highly significant increase was observed at 30th and 60th day respectively. However, HCT, MCV, MCH, MCHC and WBC counts were not altered significantly at any dose of *S. cumini*. Highly significant increase was found in platelets count at both doses after 30th. While, highly significant and significant increase was observed by SCD1 and SCD2 at 60th day respectively.

**Table 2:** In vivo effects of *Syzygium cumini* leaves extract and control on rabbit’s hematological parameters

Groups	Days	Parameters							
		Hb (g/dl)	RBC (×10^3^/mm^3^)	HCT (%)	MCV (%)	MCH (pg/cell)	MCHC (%)	WBC (×10^3^/mm^3^)	Platelets (×10^3^/mm^3^)
Control	30	9.4 ± 1.46	4.6 ± 0.67	30.8 ± 4.55	67.2 ± 2.77	21.4 ± 2.19	30.2 ± 1.64	4.8 ± 1.13	284.4 ± 64.19
	60	9.5 ± 1.29	4.5 ± 0.63	29.8 ± 4.76	68.2 ± 2.68	20.8 ± 1.64	29.4 ± 1.52	4.9 ± 1.17	296.2 ± 59.46
SCD1	30	10.4 ± 1.04	4.9 ± 0.41	32.2 ± 1.92	68.2 ± 3.35	21.2 ± 1.10	30.4 ± 1.52	4.2 ± 0.45	532.6 ± 60.84**
	60	10.6 ± 0.72	5.0 ± 0.17	31.2 ± 2.59	67.0 ± 3.16	20.6 ± 1.14	31.0 ± 1.41	4.1 ± 0.43	519.0 ± 46.56**
SCD2	30	11.5 ± 0.49*	5.8 ± 0.51*	35.2 ± 1.92	66.6 ± 3.65	20.6 ± 1.82	30.6 ± 1.14	4.7 ± 1.31	449.4 ± 54.17**
	60	11.6 ± 0.37*	5.9 ± 0.47**	35.8 ± 2.59	67.4 ± 2.30	21.2 ± 1.10	31.2 ± 0.84	4.6 ± 1.31	459.4 ± 53.15*

*n* = 10, Average values ± SEM;

SCD1: *S. cumini* dose 1; 150 mg/kg; SCD2: *S. cumini* dose 2; 500 mg/kg

*p ≤ 0.05 significant as compared to control; **p ≤ 0.005 highly significant as compared to control

*Hb* Hemoglobin, *RBC* Red blood cells, *HCT* Hematocrit, *MCV* Mean corpuscular volume, *MCH* Mean corpuscular hemoglobin, *MCHC* Mean corpuscular hemoglobin concentration, *WBC* White blood cells

Table 3 shows the activity of *S. cumini* on inhibition of platelet aggregation at two different concentrations. The methanolic extract of *S. cumini* significantly inhibited ADP and epinephrine induced platelets aggregation at 150 mg/kg concentration for 30 and 60 days. At 500 mg/kg, extract showed significant inhibition of platelets induced by AA, ADP, collagen and epinephrine in a group that received extract for 60 days. While inhibition after 30 days, was associated with only collagen and epinephrine. Moreover, Table 4 shows the presence of different phytochemical constituents such as flavonoids, phenolic constituents, terpenoids, tannins and alkaloids in methanolic leaves extract of *S. cumini* using different chemical identification tests.

**Table 3:** In vivo comparison of *S. cumini* leaves extract, aspirin and control on inhibition of platelet aggregation in rabbits. Agg RAM aggregometer was used to perform this test. Blood was taken in the ratio of 9:1 v/v in trisodium citrate (3.8%)

Groups	Days	Inhibition of platelet aggregation (%)			
		AA (500 μg/mL)	ADP (20 μmol/L)	Collagen (10 μg/mL)	Epinephrine (300 μmol/L)
Control	30	69.54 ± 8.12	41.54 ± 3.24	20.74 ± 1.54	14.65 ± 0.88
	60	76.12 ± 7.58	41.66 ± 3.68	20.65 ± 0.89	14.24 ± 0.97
SCD1	30	65.29 ± 6.21*	36.66 ± 0.54*	20.41 ± 0.75	13.45 ± 1.24
	60	57.55 ± 7.08*	37.80 ± 0.87*	17.54 ± 1.12*	13.20 ± 1.54*
SCD2	30	60.89 ± 6.29**	35.37 ± 0.70*	18.80 ± 0.98	13.98 ± 0.95
	60	52.06 ± 5.98**	33.65 ± 1.21*	16.99 ± 2.54*	13.00 ± 0.84*
Aspirin	30	43.44 ± 8.45**	30.45 ± 0.98**	12.87 ± 1.23**	12.59 ± 1.05*
	60	46.87 ± 8.99**	26.76 ± 1.25**	12.33 ± 1.01**	12.98 ± 0.90*

*n* = 10, Average values ± SEM;

SCD1: *S. cumini* dose 1; 150 mg/kg; SCD2: *S. cumini* dose 2; 500 mg/kg

*p ≤ 0.05 significant as compared to control; **p ≤ 0.005 highly significant as compared to control

*AA* Arachidonic acid, *ADP* Adenosine diphosphate

**Table 4:** Presence of phytochemical components in *S. cumini* methanolic leaves extract

S. No.	Phytochemicals	Results
1	Flavonoids	Positive^a^
2	Phenolic compounds	Positive
3	Terpenoids	Positive
4	Tannins	Positive
5	Alkaloids	Positive

^a^Presence of phytochemicals

**Fig. 1:**
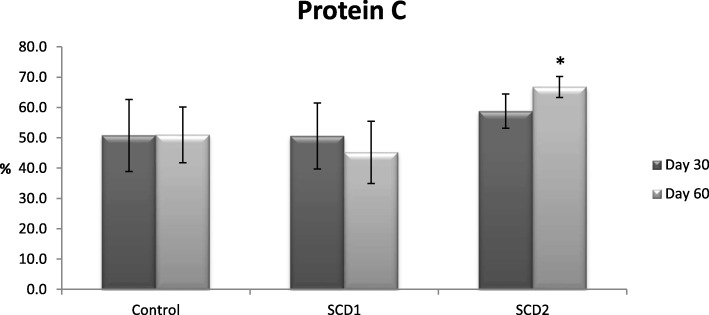
Effects of *Syzygium cumini* and control on Protein C in rabbits. Data is represented as Average values ± SEM, where *n* = 10; Two different doses of *S. cumini* were used as follows: SCD1:150 mg/kg; SCD2: 500 mg/kg, *p ≤ 0.05 significant as compared to control

**Fig. 2:**
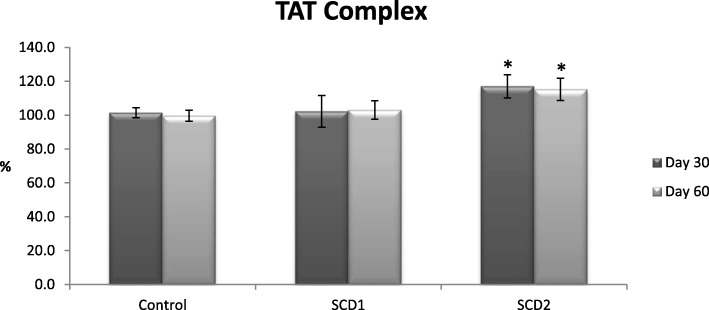
In vivo effects of *Syzygium cumini* and control on TAT complex in rabbits. Data is represented as average values ± SEM, where *n* = 10; Two different doses of *S. cumini* were used as follows: SCD1:150 mg/kg; SCD2: 500 mg/kg, *p ≤ 0.05 significant as compared to control

Figures 1 and 2 shows the effect of *S. cumini* on PC and plasma activity of TAT complexes. In Fig. 1, animals received SCD2 showed significant increase in PC level at 60th day as compare to control group. Moreover at SCD2, *S. cumini* also showed significant increase in TAT complex activity at 30th and 60th day presented in Fig. 2.

## Discussion

Danger of bleeding could be explained by BT and various coagulation tests i.e. TT, PT, aPTT and Fb levels [26]. Therefore, these tests were used to evaluate effect of *S. cumini* extract on coagulation and its potential for related cardiovascular problems.

PT and aPTT are important evaluation tests used to determine intrinsic and extrinsic pathways of coagulation [27]. It was observed in this study that aPTT at 30th and 60th day were significantly increase. It is reported that inhibition of intrinsic coagulation factors is responsible for aPTT prolongation [5, 18]. This response could be linked with the hyperlipidemic effect of *S. cumini* [28]. Significant increase in clotting factors II, VII, and X was reported due to high cholesterol diet in rabbits [29]. Increase in plasma concentration of clotting factors stimulates in response to greater catabolic rate of prothrombin [30]. Hence, we may associate that cholesterol lowering potential of *S. cumini* could be the reason of prolongation in aPTT.

SCD2 prolongs aPTT, which shows that there is inhibition of intrinsic pathway of coagulation process. It is evident that *S. cumini* contains limonene, flavonoids and phenolic acids that may prolong aPTT [30]. Results of the study showed that there is highly significant reduction in fibrinogen concentration at 30th and 60th day and these reduced levels leads to increase in bleeding time. Hence, it may be concluded that inhibition of platelet aggregation might be due to this effect of *S. cumini,* since platelets need fibrinogen to activate aggregation of platelets [31]. Due to this, inhibition of platelet aggregation increases the bleeding time. Moreover, this observed activity might be due to the presence of quercetin responsible for inhibition of platelets functions by acting on phospholipase C pathway. Presence of anthocyanins in *S. cumini* might be also responsible for inhibition of platelet aggregation [9].

Various other studies reported that PC and TAT complexes were presented as biomarker for cardiovascular and thrombotic events [30]. As *S. cumini* increased the levels of these biomarker which in turn is cause of reduce thrombin activity. This effect is similar to that of known anticoagulant drug, heparin, which might be probable reason for prolonged bleeding time. It is known that heparin potentiates synthesis of anti-thrombin III (AT-III), which ultimately, promotes complex formation with thrombin and deactivates multiple coagulation factors that increase duration of aPTT [18]. AT-III and activated form of PC play major role as natural anticoagulant. AT-III retards activated clotting factors i.e. factor Xa and thrombin (factor IIa). The increase of thrombin inhibition due to incorporation with AT-III can be estimated by TAT complex [32]. PC is also play its role in reducing thrombin and deactivate the factor VIIIa and factor Va by combining with protein S [33].

Evaluation of hematological variables could be utilized to unveil unfavorable effects of exogenous compounds i.e. plant extracts on the composition of blood in animal model [34]. Disturbance in the hematological values reflect variation in normal physiological status which also provide useful information about normal histology and histopathology of organs.

Parameters of red blood cells i.e. Hb, RBC, HCT, MCV, MCH, and MCHC were performed to evaluate the effects of *S. cumini* leaves extract in anemic state. Following administration of *S. cumini* extract, RBC and Hb levels were significantly increased, specifically at 500 mg/kg. It indicates that *S. cumini* plant extract might contain some active chemical moiety which can promote the erythropoietin secretion in stem cells. Erythropoietin is a stimulator hormone for red blood cells formation in the bone marrow [35]. This increase in RBC count improves the oxygen carrying potential of blood. Although mechanism behind this parameter is not being explored in this study, but it could be due to the phytochemical constituents that are present in this plant extract. These phytochemicals may be responsible for reduction in lipid peroxidation that ultimately prevent RBC’s haemolysis and improves its turnover. Previous study about phytochemical constituents of this plant unveiled the presence of polyphenolic compounds and flavonoids which possessed good antioxidant potential [9]. Hence, could retard the lipid peroxidation of cell membrane and haemolysis of RBSs. Platelet also plays vital role in maintenance of hemostasis. Hence low platelet counts or thrombocytopenia leads to hemorrhage or severe loss of blood [36]. Both doses of *S. cumini* showed significant increase in platelet count. It is reported that this increased platelet effect by *S. cumini* could be due to antioxidant potential effect of flavonoids present in it [8]. There was, however, significant increase in platelets count by *S. cumini* extract. This could be credited to the antioxidant activity of the flavonoids, quercetin, catechin, myricetin and rutin present which was found in methanolic leaves extract [8, 16, 36]. Antioxidant activity counters platelet oxidation and platelet dysfunction and therefore preserves platelets lifespan and increases their overall count [36]. Hence it has been reported that total flavonoid content in the methanolic leaves extract of *S. cumini* were highest than other commonly utilized parts, seeds and pulp [16].

According to pharmacochemist’s opinion, isolation of active compound(s) from methanolic extract of *S. cumini* should be carried out to identify newer anticoagulant prototypes. Moreover, thorough pharmacological studies should then be further conducted in order to determine their complete and molecular mechanism(s) of action. Also, to perform investigation of potential antithrombotic activity of isolated molecule(s) from *S. cumini* leaves.

However, extraction of plant material performed in this study was not exactly same as to the traditional approach when the interior Sindh population used water for extracts, whereas, we have used methanol due to greater pharmacological activity reported in previous studies. It was worthful to conduct in vivo studies on *S. cumini* extract and should determine adverse effects of the active constituents, pharmacokinetic properties, serum-attainable levels and diffusion in different body tissues.

## Conclusion

Hence, in the light of presence findings of this investigation it is concluded that antithrombin activity of methanolic extract of *S. cumini* leaves may be supported by its anti-inflammatory and antioxidant activities. Thus, antiplatelet effects and antithrombin activity of *S. cumini* leaves extract were found in our study. It opens a new window for future investigation on *S. cumini* leading to the development of an herbal therapeutic agent which may be of value in cardiovascular diseases.

## Data Availability

The data sets used and/or analyzed during the current study available from the corresponding author on reasonable request.

## Abbreviations

AA: Arachidonic acid
ADP: Adenosine diphosphate
aPTT: Activated partial thromboplastin time
AT-III: Anti-thrombin III
BT: Bleeding time
CAM: Complementary alternative medicines
DUHS: Dow university of health sciences
EDTA: Ethylene diamine tetra acetate
Fb: Fibrinogen
Hb: Hemoglobin
HCT: Hematocrit
MCH: Mean corpuscular hemoglobin
MCHC: Mean corpuscular hemoglobin concentration
MCV: Mean corpuscular volume
PC: Protein C
PPP: Platelets poor plasma
PRP: Platelets rich plasma
PT: Prothrombin time
RBCs: Red blood cells
SCD1: *Syzygium cumini* dose 1
SCD2: *Syzygium cumini* dose 2
SEM: Standard error mean
SPSS: Statistical package for social sciences
TAT: Thrombin antithrombin
TT: Thrombin time
WBCs: White blood cells
WHO: World health organization

## References

[1] Burns MM (2000). Alternative medicine: herbal preparations. Clin Ped Emer Med.

[2] Atchibri AOA, Brou KD, Kouakou TH, Kouadio YJ, Gnakri D (2010). Screening for antidiabetic activity and phytochemical constituents of common bean (*Phaseolus vulgaris* L.) seeds. J Medic Plant Res.

[3] Mukerjee PK, Wahil A (2006). Integrated approaches towards drug development from Ayurveda and other Indian system of medicine. J Ethnopharmacol.

[4] Yeh GY, Davis RB, Phillips RS (2006). Use of complementary therapies in patients with cardiovascular disease. Am J Cardiol.

[5] Adhyapak MS, Kachole MS (2016). Investigation of adverse effects of interactions between herbal drugs and natural blood clotting mechanism. J throm thrombol.

[6] Mann KG, Butenas S, Brumme K (2003). The dynamics of thrombin formation. Arterioscler Tromb Vasc Biol.

[7] Wang X, Hsu MY, Steinbacher TE, Monticello TM, Schumacher WA (2007). Quantification of platelet composition in experimental venous thrombosis by real-time polymerase chain reaction. Thromb Res.

[8] Bandiola TMB, Corpuz MJAT (2018). Platelet and leukocyte increasing effects of Syzygium Cumini (L.) skeels (Myrtaceae) leaves in a murine model. Pharm Anal Acta.

[9] Ruan ZP, Zhang LL, Lin YM (2008). Evaluation of the antioxidant activity of Syzygium cumini leaves. Molec.

[10] Avila-Peña D, Peña N, Quintero L, Suárez-Roca H (2007). Antinociceptive activity of *Syzygium jambos* leaves extract on rats. J Ethnopharmacol.

[11] Jain A, Sharma S, Goyal M, Dubey S, Jain S, Sahu J, Kaushik A (2010). Anti-inflammatory activity of *Syzygium cumini* leaves. Intern J Phytomed.

[12] Schoenfelder T, Warmlin CZ, Manfredini MS, Pavei LL, Réus JV, Tristão TC, Costa-Campos L (2010). Hypoglycemic and hypolipidemic effect of leaves from *Syzygium cumini* (L.) Skeels, Myrtaceae in diabetic rats. Rev Bras de Farmacog.

[13] Chagas VT, França LM, Malik S, Paes AMDA (2015). *Syzygium cumini* (L.) skeels: a prominent source of bioactive molecules against cardiometabolic diseases. Front Pharmacol.

[14] Atale N, Chakraborty M, Mohanty S, Bhattacharya S, Nigam D, Sharma M, Rani V (2013). Cardioprotective role of *Syzygium cumini* against glucose-induced oxidative stress in H9C2 cardiac myocytes. Cardiovasc Toxicol.

[15] Pekkarinen SS, Heinonen IM, Hopia AI (1999). Flavonoids quercetin, myricetin, kaemferol and (+)-catechin as antioxidants in methyl linoleate. J Sci Food Agri.

[16] Mohamed AA, Ali SI, El-Baz FK (2013). Antioxidant and antibacterial activities of crude extracts and essential oils of Syzygium cumini leaves. PLoS One.

[17] Jasprica I, Mornar A, Debeljak Ž, Smolčić-Bubalo A, Medić-Šarić M, Mayer L, Šverko V (2007). In vivo study of propolis supplementation effects on antioxidative status and red blood cells. J Ethnopharmacol.

[18] Riaz A, Khan RA (2016). Anticoagulant, antiplatelet and antianemic effects of *Punica granatum* (pomegranate) juice in rabbits. Blood Coagul Fibrinolysis.

[19] Zakaria ZA, Sani MHM, Cheema MS, Kader AA, Kek TL, Salleh MZ (2014). Antinociceptive activity of methanolic extract of Muntingia calabura leaves: further elucidation of the possible mechanisms. BMC Complement Altern Med.

[20] Li H, Cone J, Fong M, Kambayashi J, Yoshitake M, Liu Y (2005). Antiplatelet and antithrombotic activity of cilostazol is potentiated by dipyridamole in rabbits and dissociated from bleeding time prolongation. Cardiovasc Drugs Ther.

[21] McNerlan SE, Crawford VL, Stout RW (1997). Measurement of fibrinogen in frozen plasma. Thromb Res.

[22] Son DJ, Lee HW, Shin HW, Lee JJ, Yoo HS, Kim TJ, Yun YP, Hong JT (2008). Enhanced release of sphingosine-1-phosphate from hypercholesterolemic platelets: role in development of hypercholesterolemic atherosclerosis. Prostaglandins Leukot Essent Fatty Acids.

[23] Jeong YH, Hwang JY, Kim IS, Park Y, Hwang SJ, Lee SW (2010). Adding cilostazol to dual antiplatelet therapy achieves greater platelet inhibition than high maintenance dose clopidogrel in patients with acute myocardial infarction: results of the adjunctive cilostazol versus high maintenance dose clopidogrel in patients with AMI (ACCEL-AMI) study. Circ Cardiovasc Interv.

[24] Wayne PA (1998). “collection, transport and processing of blood specimens for coagulation testing and general performance of coagulation assays,” approved guideline H21-A2, 3rd edition, National Committee for Clinical Laboratory Standards.

[25] Mumtaz N, Naqvi SBS, Asghar MA, Asghar MA (2017). Assessment of antimicrobial activity of Sphaeranthus indicus L. against highly resistant pathogens and its comparison with three different antibiotics. J Dis Glob Health.

[26] Gentry PA (2004). Comparative aspects of blood coagulation. Veter J.

[27] Rao LV, Okorodudu AO, Petersen JR, Elghetany MT (2000). Stability of prothrombin time and activated partial thromboplastin time tests under different storage conditions. Clin Chim Acta.

[28] Mitropoulos KA, Esnouf MP, Meade TW (1987). Increased factor VII coagulant activity in the rabbit following diet-induced hypercholesterolaemia: evidence for increased conversion of VII to αVIIa a and higher flux within the coagulation pathway. Atherosclerosis.

[29] Miller GJ (2005). Dietary fatty acids and the haemostatic system. Atherosclerosis.

[30] Cao P, Xie P, Wang X, Wang J, Wei J, Kang WY (2017). Chemical constituents and coagulation activity of Agastache rugosa. BMC Complem Altern Med.

[31] Riaz A, Khan RA, Mirza T, Mustansir T, Ahmed M (2014). In vitro/in vivo effect of *Citrus limon.* (L. Burm. F.) juice on blood parameters, coagulation and anticoagulation factors in rabbits. Pak J Pharm Sci.

[32] Chandler WL, Velan T (2003). Estimating the rate of thrombin and fibrin generation *in vivo* during cardiopulmonary bypass. Blood.

[33] Lipe B, Ornstein DL (2011). Deficiencies of natural anticoagulants, protein C, protein S, and antithrombin. Circulation.

[34] Oyedemi SO, Adewusi EA, Aiyegoro OA, Akinpelu DA (2011). Antidiabetic and haematological effect of aqueous extract of stem bark of *Afzelia africana* (smith) on streptozotocin-induced diabetic Wistar rats. Asin Paci J Trop Biomed.

[35] Mahmoodi M, Hajizadeh M, Rashidinejad H, Asadikaram G, Khaksari M, Mirzaee M (2008). Survey of changes in complete blood count and red cell indices of whole blood incubated *in vitro* at different temperatures up to 48 hours. J Ayub Med Col Abbott.

[36] Li W, Xie R, Fan Z, Yang J, Liang W, Wu Q (2019). The contribution of oxidative stress to platelet senescence during storage. Transfusion.

